# Impact of SARS-CoV-2 variants and demographic factors on COVID-19 severity in pediatric patients

**DOI:** 10.1186/s12879-026-13987-z

**Published:** 2026-07-17

**Authors:** Thomas T. Barter, Dejerianne Ostrow, Carly Weaver, John C. Wood, David Warburton, Matthew Deardorff, Xiaowu Gai, Jennifer Dien Bard, Sindhu Mohandas

**Affiliations:** 1 Division of Infectious Disease, Children’s Hospital of Los Angeles, 4650 Sunset Blvd, Los Angeles, CA 90027 USA; 2 Keck School of Medicine, University of Southern California, 3551 Trousdale Parkway, Los Angeles, CA 90089 USA; 3 Department of Pathology and Laboratory Medicine, Children’s Hospital Los Angeles, 4650 Sunset Blvd, Los Angeles, CA 90027 USA; 4 Department of Pediatrics and Radiology, Children’s Hospital Los Angeles, 4650 Sunset Blvd, Los Angeles, CA 90027 USA; 5 Maria Fareri Children’s Hospital, Valhalla, NY USA; 6 Department of Pediatrics, Medical College of Wisconsin, Milwaukee, WI 53226 USA; 7 Mellowes Center for Genomic Sciences and Precision Medicine, Milwaukee, WI 53226 USA

**Keywords:** SARS-CoV-2, COVID-19 Severity, Pediatrics, Viral variants, Patient demographics, Health disparities, Genome sequencing

## Abstract

**Background:**

Factors that influence pediatric COVID-19 severity remain incompletely understood, particularly related to the impact of viral and host factors. Understanding the factors contributing to the severity of presentation can impact clinical approaches and management. This study examined the associations between SARS-CoV-2 variants, demographic factors, and COVID-19 severity in a large diverse pediatric population.

**Methods:**

We conducted a retrospective cohort study at a large freestanding Children’s Hospital (Children’s Hospital Los Angeles) from March 2020 through April 2024 including 14,292 patients aged 0–25 years who tested positive for SARS-CoV-2. Viral variants were determined by whole genome sequencing in 6,939 patients (48.6%). COVID-19 severity was classified as mild, moderate, or severe based on hospitalization requirements and need for respiratory support following the World Health Organization Criteria. Disease severity comparisons were made between different viral variants and demographic factors using multinomial logistic regression.

**Results:**

Among 14,292 patients, 53.8% were male with median age 5.2 years. Racial/ethnic distribution was 63.0% Hispanic/Latino, 21.4% White, 9.3% African American, and 6.3% Asian. Compared with pre-Omicron variants, Omicron was associated with increased odds of moderate (odds ratio [OR], 2.85; 95% CI, 2.31–3.52; *P* < 0.001) and severe disease (OR, 3.95; 95% CI, 3.01–5.18; *P* < 0.001). White patients had lower odds of moderate (OR, 0.81; 95% CI, 0.67–0.98) and severe disease (OR, 0.82; 95% CI, 0.64–1.05) compared with non-White patients. Age-race interactions were significant, with different severity patterns across racial groups as age increased.

**Conclusions:**

Our findings suggest that both viral variants and host demographic factors contribute meaningfully to pediatric COVID-19 severity. Understanding these interactions would support a more tailored approach to pediatric COVID-19 that considers the impact of viral variants and host demographic factors.

**Supplementary Information:**

The online version contains supplementary material available at https://doi.org/10.1186/s12879-026-13987-z.

## Background

SARS-CoV-2, the causative agent of coronavirus disease 2019 (COVID-19), has disproportionately affected certain demographic groups, with notable variations in disease severity across different populations [1–4]. While extensive research has characterized COVID-19 severity determinants in adults, pediatric-specific factors remain incompletely understood [5–6]. Most pediatric studies have been limited by small sample sizes, geographic constraints, or cross-sectional designs that cannot capture the dynamic evolution of viral variants throughout the pandemic [7–9].

Several factors complicate pediatric COVID-19 severity assessment. First, multicenter studies often introduce heterogeneity in clinical practices and environmental exposures that may obscure variant-host interactions [10–11]. Second, most pediatric research has focused on hospitalization rates rather than systematic severity classification [12]. Third, the rapid emergence of SARS-CoV-2 variants with potentially different pathogenic properties has created additional complexity in understanding disease determinants [4, 13–17].

The relationship between viral variants and demographic factors in pediatric populations remains particularly understudied. While racial and ethnic disparities in COVID-19 outcomes have been documented in adults [18], the interaction between these host factors and specific viral variants in children has not been comprehensively evaluated. Understanding these relationships is crucial for informing clinical decision-making and public health strategies tailored to pediatric populations.

We hypothesized that SARS-CoV-2 variants would demonstrate differential associations with disease severity across demographic groups in pediatric patients. To test this hypothesis, we conducted a large, single-center retrospective cohort study utilizing viral whole genome sequencing to examine the independent and interactive effects of viral variants and demographic characteristics on COVID-19 severity in children and young adults.

## Methods

### Study design and setting

This retrospective cohort study was conducted at Children’s Hospital Los Angeles, a quaternary care pediatric medical center serving a predominantly medically underserved population in Los Angeles County, California. The study was approved by the Children’s Hospital Los Angeles Institutional Review Board with a waiver of informed consent due to the retrospective nature and use of de-identified data.

### Study population

The study population included pediatric and young adult individuals aged 0–25 years who tested positive for SARS-CoV-2 by reverse transcriptase polymerase chain reaction (RT-PCR) in the Clinical Virology Laboratory at Children’s Hospital Los Angeles between March 2020 and March 2024 [19]. A total of 14,292 patients met inclusion criteria. No exclusion criteria were applied to maintain the representativeness of the cohort.

**Table 1:** Demographic features of acute COVID patients included in the study

Age (years)	Mild (*N* (%))	Moderate (*N* (%))	Severe (*N* (%))	Total (*N* (%))
0–5	5603 (82.8%)	858 (12.7%)	305 (4.5%)	6766 (47.4%)
6–12	3435 (89.3%)	318 (8.3%)	92 (2.4%)	3845 (26.9%)
13–17	1969 (89.3%)	144 (6.6%)	91 (4.1%)	2204 (15.4%)
18–25	1321 (89.5%)	99 (6.7%)	56 (3.8%)	1477 (10.3%)
Sex				
Male	6550 (85.1%)	828 (10.8%)	320 (4.1%)	7689 (53.8%)
Female	5763 (87.6%)	591 (9.0%)	225 (3.4%)	6579 (46.0%)
Race				
African American	525 (77.4%)	106 (15.7%)	47 (6.9%)	678 (9.3%)
Asian	363 (78.9%)	66 (14.4%)	31 (6.7%)	460 (6.3%)
Hispanic	3597 (78.6%)	710 (15.5%)	269 (5.9%)	4576 (63.0%)
White	1271 (81.9%)	202 (13.0%)	78 (5.1%)	1551 (21.4%)
Viral Variant available	6108 (88.0%)	573 (8.3%)	258 (3.7%)	6939 (48.6%)
Ancestral	1653	96	34	1783 (25.70%)
Delta	497	28	12	537 (7.74%)
Epsilon	682	24	6	712 (10.26%)
nVoC	972	46	21	1039 (14.97%)
Omicron	2276	378	185	2839 (40.91%)

Long COVID patients were not part of the above cohort; they were patients who were seen in the Long COVID clinic several weeks to months after their acute infection and did not have COVID testing done at our hospital. Patients with MIS-C also are a separate cohort of patients hospitalized for MIS-C 4 to 6 weeks after acute infection and mostly did not have positive SARS-CoV-2 test at the time of diagnosis. Only 26 of 218 patients with MIS-C had a positive SARS-CoV-2 PCR on admission.

### Data collection

Clinical and demographic data were extracted from the electronic health record using the Vigilanz reporting system. Data elements included age, sex, race, ethnicity, testing dates, hospitalization status, oxygen support requirements, mechanical ventilation needs, and clinical outcomes. Race was categorized as White, African American, Asian, Hispanic/Latino, Native American/Alaska Native, Native Hawaiian/Pacific Islander, or multiple/other races. Hispanic/Latino was retained as a racial category to reflect its inclusion as a self-reported option in the hospital admissions system. Ethnicity was classified as Hispanic/Latino or non-Hispanic/Latino based on patient self-report or parental report for minors. Comorbidity data were extracted by querying patient diagnostic records for condition-specific key words and subsequently classified into five categories: respiratory disease, neurological conditions, cardiovascular disease, immunodeficiency, and obesity.

### SARS-CoV-2 whole genome sequencing

Specimens with RT-PCR cycle threshold (Ct) values less than 30 were selected for whole genome sequencing (WGS) to ensure adequate viral RNA concentration for reliable sequencing results. Sequencing was performed using either the Paragon Genomics CleanPlex SARS-CoV-2 Research and Surveillance Next-Generation Sequencing Panel or the AmpliSeq SARS-CoV-2 Insight Research Assay on Ion Torrent Genexus platform, following previously described protocols [19–20].

Viral lineages were classified according to World Health Organization (WHO) nomenclature and variant designation criteria. Variants without official WHO designation were categorized as “non-variants of concern” (nVoC). To ensure statistical power for comparative analyses, viral lineages with fewer than 10 patients were excluded from variant-specific analyses.

### COVID-19 severity classification

Disease severity was classified using a modified WHO severity assessment framework, focusing on hospitalization requirements and respiratory support needs. The classification system included three categories:

- Mild disease: Asymptomatic or outpatient management for mild symptoms without hospitalization specifically attributable to COVID-19.
- Moderate disease: Hospitalization for COVID-19 management without requirement for mechanical ventilation or vasopressor support.
- Severe disease: Hospitalization with requirement for mechanical ventilation, vasopressor support, or intensive care unit admission.

To filter for hospitalizations related to COVID-19, we searched admitting diagnoses for keywords that would indicate that the reason for hospital visit was COVID-19 related. In addition, we correlated this with the medications used (Remdesivir and steroids) to ensure that worsening severity was more likely related to COVID-19 than another condition. Hospitalizations unrelated to COVID-19 (such as trauma, elective procedures, or other primary diagnoses) were not considered in severity classification. Cases with discordant classification were reviewed by two independent investigators, with consensus reached through discussion.

### Statistical analysis

Basic summary statistics were calculated for all study variables. Categorical variables (such as sex, race, and viral variant) are reported as counts and percentages, while continuous variables (such as age) are reported as medians with interquartile ranges.

To examine which patient factors were associated with more severe COVID-19 illness, we used multinomial logistic regression — a statistical method that estimates the likelihood of belonging to one outcome group (moderate or severe disease) compared to a reference group (mild disease). Results are expressed as odds ratios, where a value greater than 1 indicates increased odds of more severe disease.

The analysis accounted for patient age, sex, race, ethnicity, and infecting viral variant. We also tested whether certain combinations of these factors interacted with one another — for example, whether the relationship between age and disease severity differed across racial groups or viral variants. Wald test was performed to determine significance for each interaction term. Non-significant interaction terms were removed from the final models, retaining only those with meaningful contributions (*P* < 0.10 for interactions, *P* < 0.05 for individual predictors).

To ensure all possible comparisons between viral variants and demographic groups were captured, the reference group was systematically rotated — for instance, each viral variant was used as the baseline in turn, allowing head-to-head comparisons across all variant pairs.

Because multiple group comparisons were made simultaneously, we applied the Benjamini-Hochberg correction to reduce the risk of false-positive findings, with a false discovery rate threshold of 5%. Missing demographic data were minimal (less than 5% of observations) and occurred at random, so no imputation was required.

All statistical analyses were performed using R version 4.3.0 [21] with the nnet package [22] version 7.3–19 for multinomial logistic regression modeling. Two-sided P values less than 0.05 were considered statistically significant.

## Results

### Cohort characteristics

Among 14,292 patients with laboratory-confirmed SARS-CoV-2 infection, the median age was 5.2 years (interquartile range, 1.8–12.4 years), and 7,689 (53.8%) were male. The cohort demonstrated substantial demographic diversity, with 4,576 (63.0%) Hispanic/Latino patients, 1,551 (21.4%) White patients, 678 (9.3%) African American patients, and 460 (6.3%) Asian patients. Children aged 0–5 years comprised 6,766 (47.4%) of the cohort, representing a higher proportion than national pediatric demographics (30.9%) (Table 1).

Disease severity was distributed as 12,328 patients (86.2%) with mild disease, 1,419 (9.9%) with moderate disease, and 544 (3.8%) with severe disease. The youngest age group (0–5 years) demonstrated slightly higher rates of moderate and severe disease (17.2%) compared with older age groups: 6–12 years (10.7%), 13–17 years (10.7%), and 18–25 years (10.5%) (Table 1, see Additional file 1).

### SARS-CoV-2 whole genome sequencing results

Whole genome sequencing was successfully performed on 6,939 specimens (48.6% of total cohort), representing patients with RT-PCR Ct values less than 30. The temporal distribution of viral variants demonstrated clear epidemiologic waves, with multiple variants circulating simultaneously during the pre-Omicron period and Omicron achieving dominance following its emergence in late 2021. The variant timeline at our institution showed high concordance with larger national surveillance data for the duration and succession of major variants including Alpha, Delta, and Omicron [see Additional file 2].

### Associations between demographic factors and disease severity

#### Age and sex effects

Multinomial logistic regression analysis of 14,292 patients revealed that male patients demonstrated statistically significantly higher odds of moderate disease (odds ratio [OR], 1.233) and severe disease (OR, 1.252) compared with female patients. A clear inverse age relationship was observed, with younger patients demonstrating statistically significantly higher odds of moderate and severe disease compared with older patients across the pediatric and young adult age range [see Additional file 3].

### Racial and ethnic disparities

Among the 7,286 patients included in this analysis, White patients exhibited the highest rates of mild disease and lowest rates of moderate and severe disease. Compared with non-White patients, White patients had lower odds of moderate disease (OR, 0.81) and severe disease (OR, 0.82), though these differences did not achieve statistical significance in the overall cohort [see Additional files 4]. When comparing individual racial groups, African American, Asian, and Hispanic patients demonstrated similar severity rates when compared with each other, with no statistically significant differences observed between non-White racial groups.

### SARS-CoV-2 variant associations with disease severity

Viral variant was examined as a predictor of COVID-19 disease severity among 6,939 patients. Omicron variant infection was associated with significantly increased odds of moderate disease (OR, 2.85; *P* < 0.001) and severe disease (OR, 3.95; *P* < 0.001) compared with all pre-Omicron variants. All pre-Omicron variants (ancestral, Delta, Epsilon, nVoC) demonstrated similar severity distributions with no statistically significant differences between them [see Additional file 5]. Alpha variant was excluded from comparative analyses due to insufficient sample size after stratification by severity categories.

**Fig. 1:**
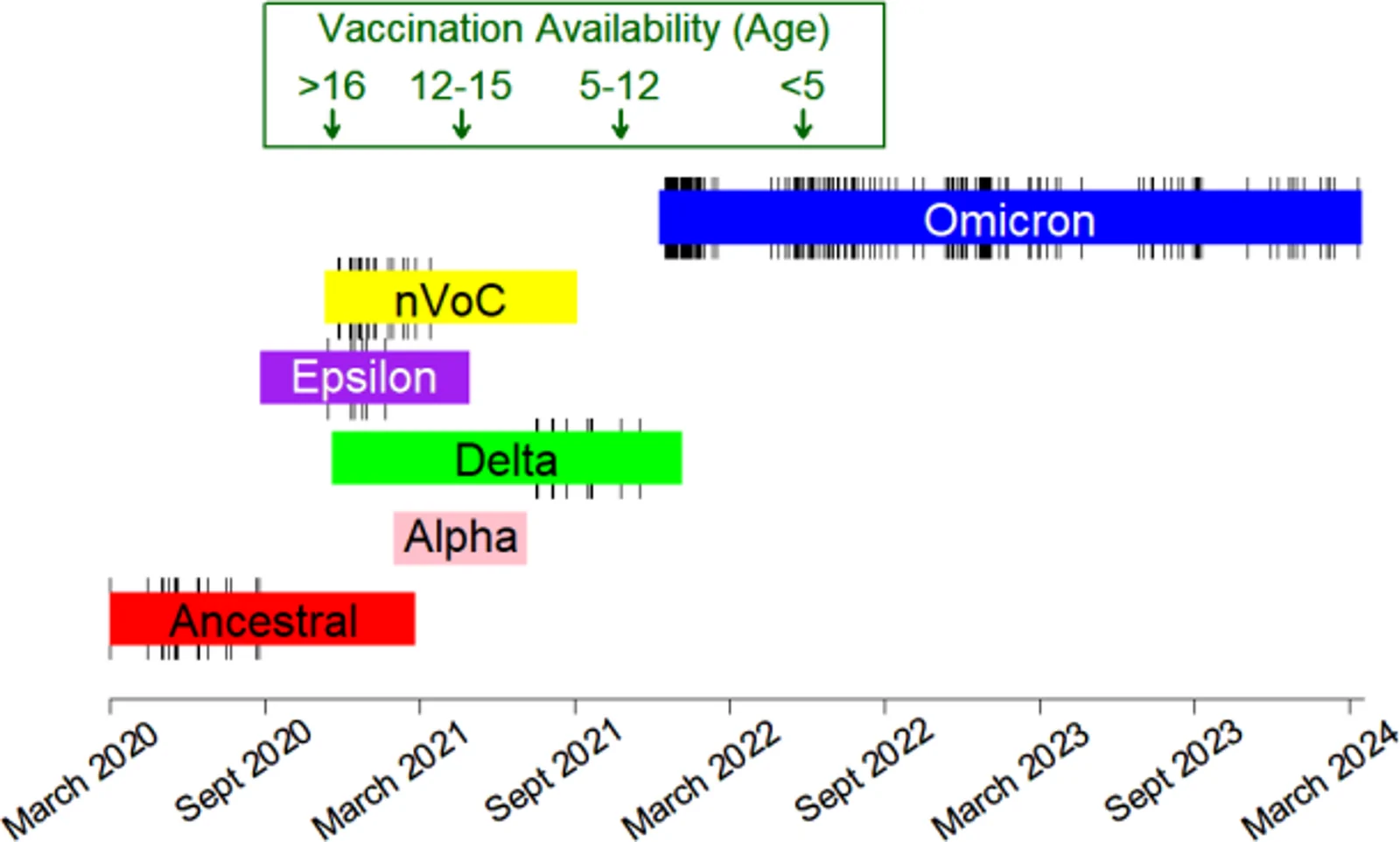
COVID-19 variant for each severe case. Viral WGS was used to determine COVID-19 variant for each severe patient. Vaccination availability is also indicated for each age group

Temporal analysis of severe cases revealed apparent spikes in severe disease at the onset of both Delta and Omicron periods. However, viral genome sequencing data demonstrated that most severe cases during the apparent “Delta surge” were actually attributed to nVoC rather than Delta variant, highlighting the importance of molecular characterization rather than temporal assignment of variants (Fig. 1). Interestingly, all severe Delta cases from our hospital came in the latter half of the Delta period.

### Age-variant and age-race interaction effects

**Fig. 2:**
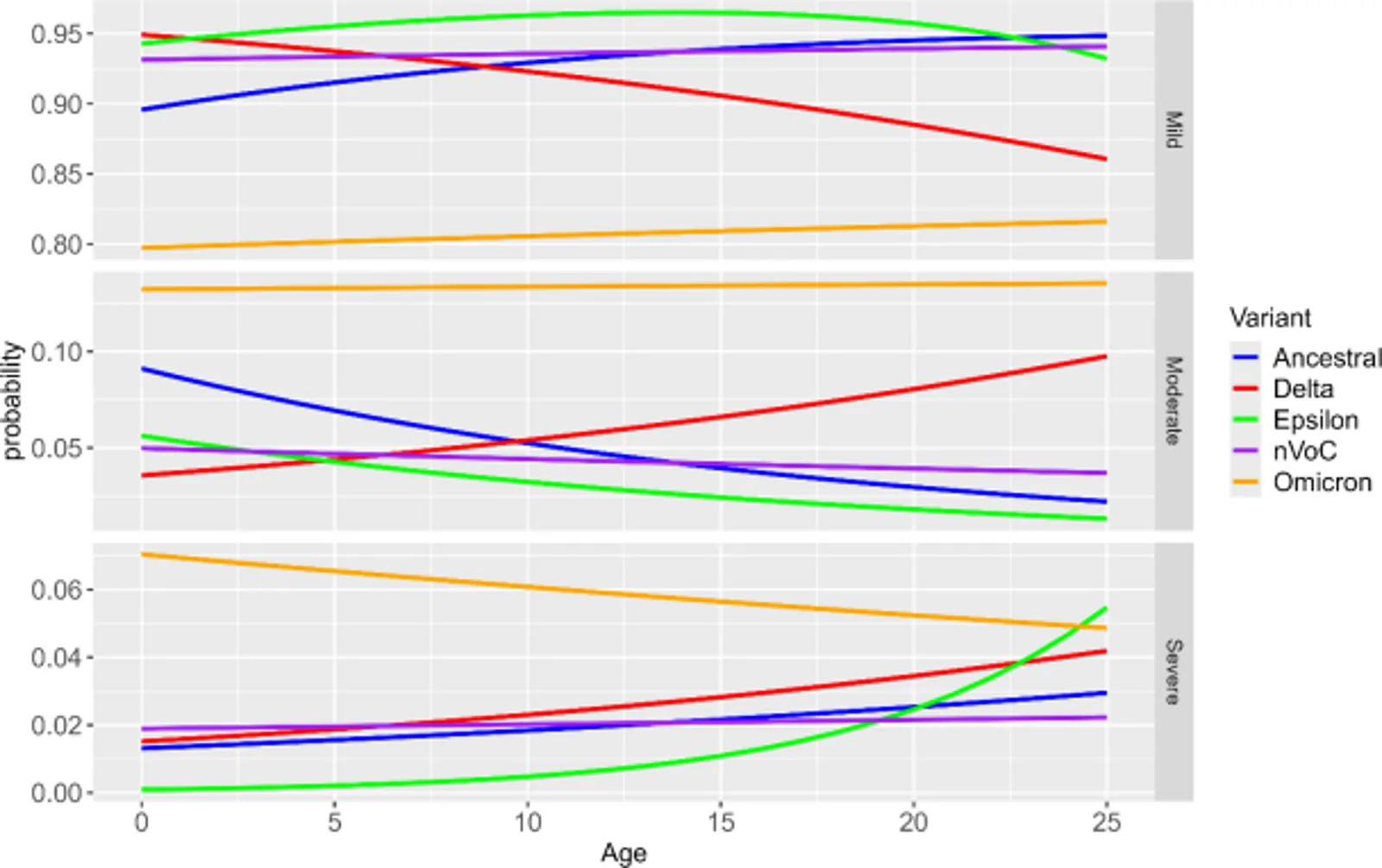
Multinomial logistic regression predicting severity from COVID-19 variants and age. Comparison of COVID-19 variant with age and their effect on disease severity across ages 0–25. Each line represents the probability of falling within each disease severity category given COVID-19 variant and age

Analysis of age-variant interactions among 6,939 patients revealed that Delta variant exhibited unique age-related severity patterns. For Delta infections, both moderate and severe disease frequencies increased with patient age, contrasting with the overall inverse age-severity relationship observed across the entire cohort (Fig. 2). Epsilon appears to have a stark change in severe disease state likelihood as age increases, but the small sample size for severe cases with Epsilon makes this finding misleading. Alpha variant was excluded from interaction analyses due to absence of severe cases in the Alpha-infected population.

**Fig. 3:**
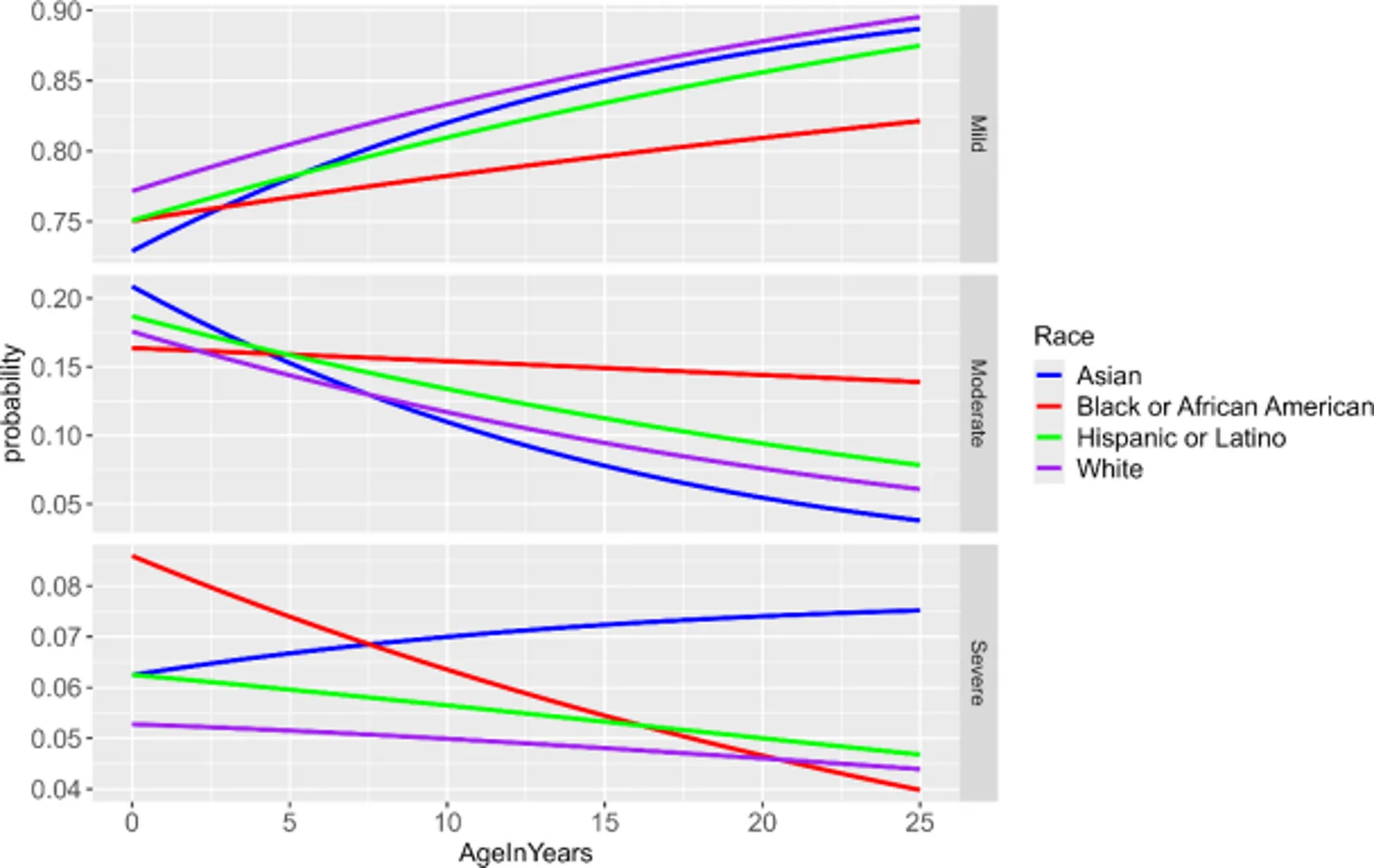
Multinomial logistic regression predicting severity from race and age. Comparison of race with age and their effect on disease severity across ages 0–25. Each line represents the probability of falling within each disease severity category given race and age

Age-race interaction analysis demonstrated that each racial group exhibited distinct age-related severity patterns (Fig. 3). Native American/Alaska Native and Native Hawaiian/Pacific Islander populations were excluded from this analysis due to insufficient representation across all severity categories. Hispanic/Latino and White patients both demonstrated decreased severity odds with increasing age. Asian patients showed decreased moderate disease odds with age but slight increases in severe disease odds at older ages. African American patients exhibited relatively consistent moderate disease rates across ages but demonstrated steep declines in severe disease odds with increasing age.

### Ethnicity-variant interaction effects

**Fig. 4:**
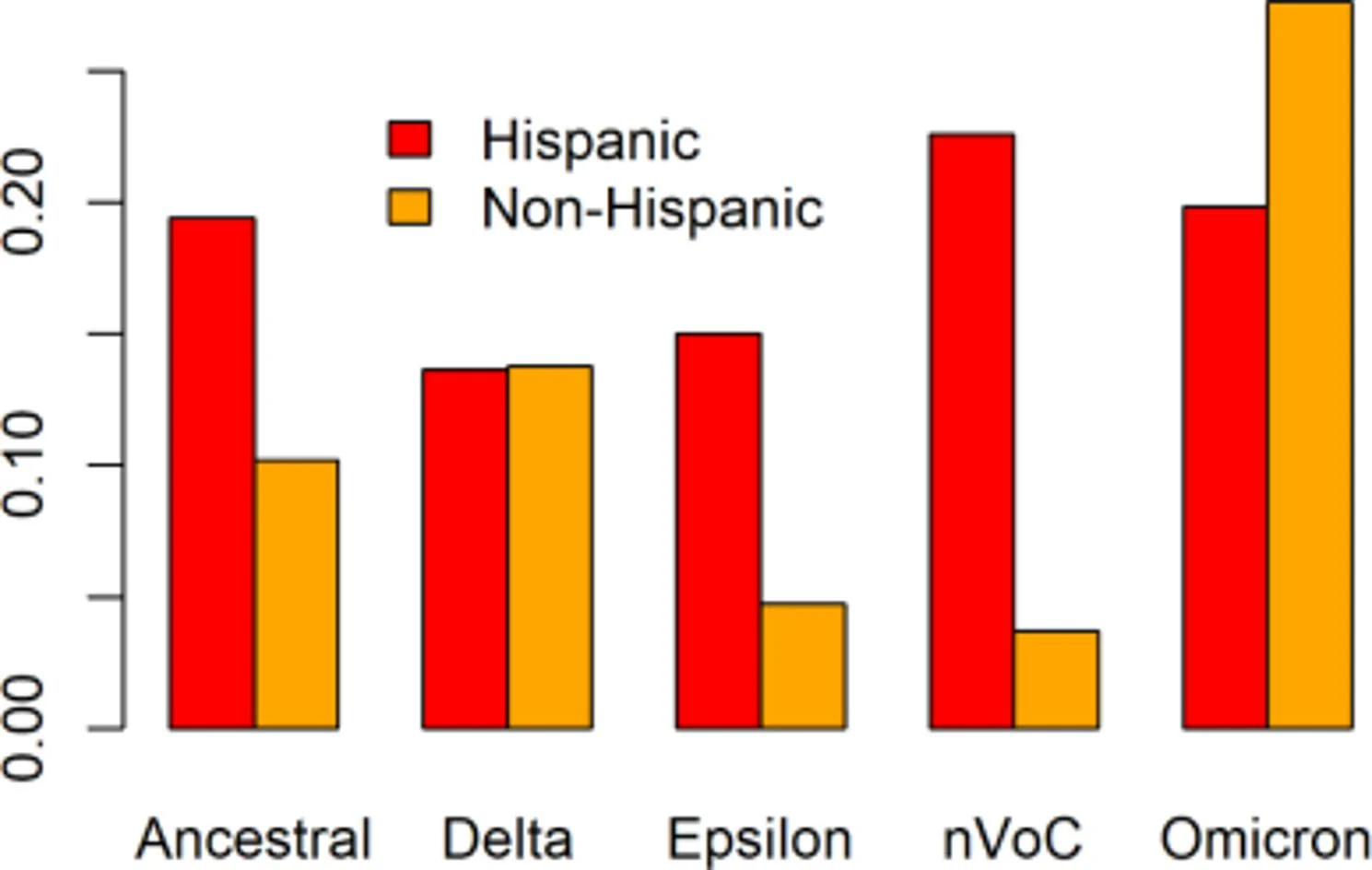
Comparison of Ethnicity-specific on COVID-19 variants predicting disease severity. Comparing non-mild cases (moderate and severe) for each variant and ethnicity combination. The Y-axis denotes the percentage of patients within each Ethnic group that presented moderate or severe disease. Moderate and severe were summed together to generate frequencies seen in the figure

Ethnicity-specific analysis of variant effects among 2,904 patients revealed significant differences in severity patterns between Hispanic/Latino and non-Hispanic/Latino populations across viral variants (Fig. 4). The Delta variant was the only variant showing similar frequencies of moderate and severe disease between ethnic groups. The Ancestral and nVoC variants were associated with higher disease severity in Hispanic/Latino patients compared to non-Hispanic/Latino patients (*p* = 0.04 and *p* < 0.001, respectively). In contrast, the Omicron variant was associated with significantly higher severity in non-Hispanic/Latino patients compared to Hispanic/Latino patients (*p* < 0.001). Notably, Hispanic/Latino patients showed no significant change in severity during the Omicron period relative to earlier variants (*p* = 0.43), whereas non-Hispanic/Latino patients experienced a statistically significant increase in severity during Omicron compared to preceding variants (*p* < 0.001).

### Comorbidities effects

Comorbidities were identified from ICD-10-CM diagnostic codes recorded in the electronic health record and categorized into five clinically meaningful groups: respiratory disease, neurological conditions, cardiovascular disease, immunodeficiency, and obesity. Multinomial logistic regression among 12,508 patients revealed that each comorbidity category was independently associated with a significantly increased likelihood of severe COVID-19 after adjustment for age and sex [See additional files 6 and 7]. The distribution of comorbidities remained largely stable across variant periods. When comorbidities were incorporated alongside race in the model among 6,709 patients, the association between race and COVID-19 disease severity was attenuated, suggesting that comorbidity burden accounts for a meaningful portion of the observed racial differences in disease severity [see Additional file 8]. Differences between the univariable and multivariable models are likely attributable to the disproportionate reduction in sample size across predictor categories resulting from listwise deletion of incomplete cases.

### Multivariable model

When all variables were incorporated into a multivariable model among 2,904 patients, findings were largely consistent with those observed in the univariable analyses. Viral variant remained a significant predictor of disease severity, with the Omicron variant associated with a significantly higher likelihood of severe disease. Increasing age was associated with decreased severity, and each comorbidity category retained an independent and significant association with disease severity. While race did not reach statistical significance in the multivariable model, the directional trend is no longer seen relative to the univariable analyses, with White patients demonstrating slightly higher rates of moderate and severe disease. The convergence of results across both modeling approaches supports the robustness of these variables as predictors of COVID-19 disease severity.

A notable limitation of the multivariable model is the substantial reduction in sample size overall and disproportionate reduction in sample size across predictor categories resulting from listwise deletion of cases with missing data across variables. The use of a more robust dataset with minimal missing data, including variables we were unable to collect in this study (such as vaccination status, socioeconomic indicators, detailed comorbidity data, and more complete race documentation), would improve the precision of future analyses and better define the relative contribution of each factor to disease severity.

### Multisystem inflammatory syndrome in children (MIS-C)

Among 218 patients with confirmed MIS-C diagnoses between July 2020 and February 2024, only 26 (11.9%) had concurrent positive SARS-CoV-2 RT-PCR results permitting direct viral variant determination. The most frequently identified variant in these confirmed cases was nVoC, representing 19 of 26 cases. Within the nVoC category, B lineage demonstrated disproportionate representation, accounting for 12 of 19 nVoC cases.

Given the typical 4–6 week delay between SARS-CoV-2 infection and MIS-C presentation, estimated variant attribution was performed based on diagnosis timing and institutional variant circulation patterns. This analysis suggested that the majority of MIS-C cases were associated with pre-Omicron era variants, with notably lower representation during the Omicron-predominant period despite Omicron infections comprising approximately 40% of the overall sequenced cohort [see Additional file 9].

### Post-acute sequelae of SARS-CoV-2 (Long COVID)

Of the 192 Long COVID cases at CHLA between July 2020 and December 2024, Long COVID diagnoses were distributed relatively uniformly across all variant periods through early 2025, with notable decreases during off season months [see Additional file 10]. Unlike MIS-C, which demonstrated clear temporal clustering in the pre-Omicron era, long COVID cases showed no apparent variant-specific concentration, suggesting that risk of post-acute sequelae may be independent of specific viral variant characteristics. This differs from the decrease in Long COVID incidence during the Omicron era seen in adults [23].

## Discussion

This large pediatric and young adult cohort study reveals complex interactions between SARS-CoV-2 variants and demographic factors in determining COVID-19 severity. Our findings challenge conventional understanding of variant severity patterns and highlight important disparities in pediatric and young adult COVID-19 outcomes.

### Principal findings and clinical implications

The most striking finding was the association between Omicron variant infection and increased moderate and severe disease in our pediatric and young adult population, with odds ratios of 2.85 and 3.95, respectively, compared with pre-Omicron variants. This contrasts sharply with adult literature demonstrating milder Omicron disease severity [7]. Several mechanisms may explain this apparent paradox. First, the widespread availability of home testing during the Omicron era likely resulted in significant ascertainment bias, with mild cases disproportionately managed at home rather than presenting to healthcare facilities. Second, as a quaternary referral center, our institution may have received a selected population of more severe Omicron cases. Third, age-related differences in immune response and viral pathophysiology may render Omicron relatively more virulent in pediatric and young adult populations compared with adult populations. Lastly, pediatric and young adult vaccine uptake (10%-60% varying with age) when Omicron was introduced was considerably lower than adult vaccine uptake (80%) in the greater Los Angeles area [24].

The observed racial disparities, with non-White patients experiencing higher odds of moderate and severe disease, align with broader health equity concerns documented throughout the pandemic [1, 25]. However, the presence of significant age-race interactions, combined with the role of comorbidity burden, suggests that demographic risk factors cannot be interpreted in isolation. The differential age-related severity patterns across racial groups likely reflect complex interplay between biological susceptibility, environmental exposures, healthcare access, and social determinants of health.

Our findings regarding male predominance in severe disease align with established literature across age groups [26]. However, the inverse relationship between age and severity within our pediatric and young adult cohort differs from adult studies, likely reflecting the restriction of our population to ages 0–25 years. The ethnicity-variant interactions we observed, particularly the differential Omicron impact on Hispanic versus non-Hispanic patients, represent novel findings that warrant replication in other populations. Importantly, this differential impact appears to be driven by increased vulnerability to severe disease among non-Hispanic patients during the Omicron period, rather than relative protection among Hispanic patients, as the distribution of moderate and severe cases remained consistent across all variant periods in the Hispanic/Latino population but differed significantly in non-Hispanic/Latino patients.

As expected, each comorbidity category was independently associated with significantly increased odds of moderate or severe COVID-19, though the magnitude of this association varied across comorbidity groups, consistent with prior literature [27, 28]. While mild variation in comorbidity prevalence was observed across variant periods, these differences were not sufficient to explain the severity differences seen between variants, suggesting that comorbidity burden was not a primary driver of variant-specific severity patterns.

### Limitations

Several important limitations must be considered when interpreting these findings. First, our single-center design at a quaternary care facility may limit generalizability and introduce referral bias toward more severe cases. The safety-net hospital status of our institution serves a predominantly medically underserved population, which may not represent broader pediatric and young adult demographics.

Second, SARS-CoV-2 WGS was restricted to specimens with cycle threshold values less than 30, which may have introduced selection bias toward cases with higher viral loads. This threshold, while ensuring sequencing success, may have excluded cases with lower viral burden that could have different severity patterns.

Third, the dramatic increase in home testing availability during the Omicron era creates significant ascertainment bias. Many mild Omicron cases likely never presented to healthcare facilities, artificially inflating the apparent severity of hospital-identified cases. This limitation is particularly relevant to our Omicron severity findings and emphasizes the need for population-based studies that capture community cases. Additionally, universal admission testing protocols enforced at our institution through May 2023 may have increased the identification of mild or asymptomatic cases during this period, potentially influencing severity estimates across variant periods.

Fourth, we cannot establish causality between variants and severity outcomes due to the observational design. Temporal confounding with vaccination rollout, treatment protocol evolution, and changing healthcare-seeking behaviors may have influenced our results.

Fifth, vaccination status could not be systematically ascertained for all patients due to limitations in inclusion of vaccination data in our electronic health record and was therefore excluded as a potential exploratory variable. This represents a notable limitation, as vaccination status may confound the relationships observed between demographic factors, comorbidities, and COVID-19 disease severity.

### Clinical and public health implications

These findings have several important clinical implications. Clinicians caring for pediatric and young adult patients should consider both variant type and demographic characteristics when assessing COVID-19 severity risk, recognizing that these factors interact in complex ways. The apparent increased severity associated with Omicron in pediatric and young adult patients, suggests continued vigilance is warranted during Omicron-predominant periods.

From a public health perspective, the persistent racial disparities observed across variants underscore the need for targeted interventions addressing social determinants of health and healthcare access barriers in communities of color. The variant-ethnicity interactions suggest that pandemic response strategies may need to be tailored not only to demographic groups but also to circulating viral strains.

### Future research directions

Several research priorities emerge from this study. First, validation of our Omicron severity findings in other pediatric and young adult populations is essential, ideally through studies that capture community cases to minimize ascertainment bias. Second, investigation of biological mechanisms underlying variant-demographic interactions could inform targeted therapeutic approaches. Third, longitudinal studies examining the evolution of these relationships as new variants emerge will be crucial for pandemic preparedness.

## Conclusions

In this large, diverse pediatric and young adult cohort, SARS-CoV-2 variants and demographic characteristics demonstrated significant and complex interactions in determining COVID-19 severity. While our finding of increased Omicron severity requires validation, the demonstrated variant-demographic interactions highlight the importance of personalized risk assessment in pediatric COVID-19 care. These results underscore the continued need for targeted public health interventions addressing persistent health disparities and the importance of comprehensive surveillance systems that can capture the full spectrum of disease severity across all populations.

## Supplementary Information

Below is the link to the electronic supplementary material.

Supplementary Material 1

Supplementary Material 2

Supplementary Material 3

Supplementary Material 4

Supplementary Material 5

Supplementary Material 6

Supplementary Material 7

Supplementary Material 8

Supplementary Material 9

Supplementary Material 10

## Data Availability

The datasets used and/or analyzed during the current study are available from the corresponding author on reasonable request. The viral WGS data is available at Zenodo repository, https://zenodo.org/records/15785663.

## Abbreviations

RT-PCR: Reverse Transcriptase Polymerase Chain Reaction
Ct: Cycle threshold
WHO: World Health Organization
nVoC: Non–variants of concern
OR: Odds ratio
