# Supplementary material for: Impact of SARS-CoV-2 variants and demographic factors on COVID-19 severity in pediatric patients
Source: BMC Infect Dis. 2026 Jul 17;26:1804. doi: 10.1186/s12879-026-13987-z (PMC13660046; doi:10.1186/s12879-026-13987-z)
Supplement: Supplementary file 1 [file 12879_2026_13987_MOESM10_ESM.docx]

**Estimated COVID-19 variant for each Long COVID patient.** Patient reported date of COVID-19 infection was used as an estimator for COVID-19 variant for each Long COVID patient. Vaccination availability is also plotted for each age group. Each line is drawn to intersect all possible variants and does not convey any quantitative information.
