# Supplementary material for: Impact of SARS-CoV-2 variants and demographic factors on COVID-19 severity in pediatric patients
Source: BMC Infect Dis. 2026 Jul 17;26:1804. doi: 10.1186/s12879-026-13987-z (PMC13660046; doi:10.1186/s12879-026-13987-z)
Supplement: Supplementary file 3 [file 12879_2026_13987_MOESM2_ESM.docx]

**Timescale of COVID-19 Variants.** Each bar represents the time of circulation for each COVID-19 variant between March 2020 and March 2024 at CHLA.
