# Supplementary material for: Impact of SARS-CoV-2 variants and demographic factors on COVID-19 severity in pediatric patients
Source: BMC Infect Dis. 2026 Jul 17;26:1804. doi: 10.1186/s12879-026-13987-z (PMC13660046; doi:10.1186/s12879-026-13987-z)
Supplement: Supplementary file 5 [file 12879_2026_13987_MOESM4_ESM.docx]

|  | **Moderate** | | |
| --- | --- | --- | --- |
|  | **Asian** | **Black or African American** | **Hispanic or Latino** |
| **Asian** |  |  |  |
| **Black or African American** | 1.11 ± 0.34 (0.536) |  |  |
| **Hispanic or Latino** | 1.09 ± 0.27 (0.556) | 0.98 ± 0.22 (0.842) |  |
| **White** | 0.87 ± 0.30 (0.384) | 0.79 ± 0.26 (0.067) | 0.81 ± 0.17 (0.012) |
|  | **Severe** | | |
| **Asian** |  |  |  |
| **Black or African American** | 1.05 ± 0.47 (0.842) |  |  |
| **Hispanic or Latino** | 0.88 ± 0.39 (0.507) | 0.84 ± 0.32 (0.275) |  |
| **White** | 0.72 ± 0.43 (0.136) | 0.69 ± 0.38 (0.049) | 0.82 ± 0.26 (0.136) |

Odds ratio of increasing in severity when comparing self-reported Race with corresponding p-values included
