# Supplementary material for: Impact of SARS-CoV-2 variants and demographic factors on COVID-19 severity in pediatric patients
Source: BMC Infect Dis. 2026 Jul 17;26:1804. doi: 10.1186/s12879-026-13987-z (PMC13660046; doi:10.1186/s12879-026-13987-z)
Supplement: Supplementary file 6 [file 12879_2026_13987_MOESM5_ESM.docx]

|  | **Moderate** | | | |
| --- | --- | --- | --- | --- |
|  | **Ancestral** | **Delta** | **Epsilon** | **nVoC** |
| **Ancestral** |  |  |  |  |
| **Delta** | 0.97 ± 0.43 (0.890) |  |  |  |
| **Epsilon** | 0.61 ± 0.46 (0.031) | 0.62 ± 0.56 (0.098) |  |  |
| **nVoC** | 0.81 ± 0.36 (0.265) | 0.84 ± 0.48 (0.477) | 1.35 ± 0.50 (0.248) |  |
| **Omicron** | 2.86 ± 0.24 (<0.001) | 2.95 ± 0.40 (<0.001) | 4.72 ± 0.42 (<0.001) | 3.51 ± 0.32 (<0.001) |
|  | **Severe** | | | |
| **Ancestral** |  |  |  |  |
| **Delta** | 1.17 ± 0.67 (0.637) |  |  |  |
| **Epsilon** | 0.43 ± 0.88 (0.056) | 0.36 ± 0.99 (0.045) |  |  |
| **nVoC** | 1.05 ± 0.56 (0.861) | 0.84 ± 0.48 (0.764) | 2.45 ± 0.91 (0.054) |  |
| **Omicron** | 3.95 ± 0.37 (<0.001) | 3.37 ± 0.59 (<0.001) | 9.24 ± 0.82 (<0.001) | 3.76 ± 0.46 (<0.001) |

Odds ratio of increasing in severity when comparing different COVID-19 variants with corresponding p-values included
