# Supplementary material for: Impact of SARS-CoV-2 variants and demographic factors on COVID-19 severity in pediatric patients
Source: BMC Infect Dis. 2026 Jul 17;26:1804. doi: 10.1186/s12879-026-13987-z (PMC13660046; doi:10.1186/s12879-026-13987-z)
Supplement: Supplementary file 7 [file 12879_2026_13987_MOESM6_ESM.docx]

|  | Moderate | Severe |
| --- | --- | --- |
| Respiratory | 18.89 (<0.001) | 14.61 (<0.001) |
| Neurological | 5.28 (<0.001) | 14.86 (<0.001) |
| Cardiovascular | 5.73 (<0.001) | 24.29 (<0.001) |
| Immunocompromised | 12.96 (<0.001) | 11.99 (<0.001) |
| Obesity | 5.79 (<0.001) | 15.87 (<0.001) |

Odds ratios and p-values for each comorbidity’s impact on increasing disease state from mild to moderate or severe.
