# Supplementary material for: Impact of SARS-CoV-2 variants and demographic factors on COVID-19 severity in pediatric patients
Source: BMC Infect Dis. 2026 Jul 17;26:1804. doi: 10.1186/s12879-026-13987-z (PMC13660046; doi:10.1186/s12879-026-13987-z)
Supplement: Supplementary file 8 [file 12879_2026_13987_MOESM7_ESM.docx]

|  | Mild (N (%)) | Moderate (N (%)) | Severe (N (%)) |
| --- | --- | --- | --- |
| Total | 12328 (86.3%) | 1419 (9.9%) | 544 (3.8%) |
| Respiratory | 204 (28.7%) | 385 (54.1%) | 122(17.2%) |
| Neurological | 65 (41.7%) | 45 (28.8%) | 46 (29.5%) |
| Cardiovascular | 99 (36.1%) | 73 (26.6%) | 102 (37.2%) |
| Immunocompromised | 60 (31.1%) | 98 (50.8%) | 35 (18.1%) |
| Obesity | 58 (39.7%) | 44 (30.1%) | 44 (30.1%) |

Cohort breakdown for each comorbidity group and disease severity.
