# Supplementary material for: Impact of SARS-CoV-2 variants and demographic factors on COVID-19 severity in pediatric patients
Source: BMC Infect Dis. 2026 Jul 17;26:1804. doi: 10.1186/s12879-026-13987-z (PMC13660046; doi:10.1186/s12879-026-13987-z)
Supplement: Supplementary file 9 [file 12879_2026_13987_MOESM8_ESM.docx]

| Variants | Moderate | Severe |
| --- | --- | --- |
| Ancestral | 1.00 | 1.00 |
| Delta | 1.22 (0.594) | 4.147 (0.022) |
| Epsilon | 0.976 (0.960) | 1.333 (0.796) |
| nVoC | 0.440 (0.081) | 1.453 (0.597) |
| Omicron | 1.465 (0.128) | 4.665 (0.002) |
| Race |  |  |
| White | 1.00 | 1.00 |
| Asian | 0.812 (0.442) | 1.060 (0.872) |
| Black or African American | 0.819 (0.403) | 0.954 (0.887) |
| Hispanic or Latino | 0.843 (0.296) | 0.656 (0.073) |
|  |  |  |
| Age | 0.945 (<0.001) | 0.915 (<0.001) |
| Comorbidities |  |  |
| Respiratory | 10.100 (<0.001) | 12.265 (<0.001) |
| Neurological | 8.970 (<0.001) | 32.317 (<0.001) |
| Cardiovascular | 5.858 (<0.001) | 42.877 (<0.001) |
| Immunodeficiency | 32.136 (<0.001) | 22.461 (<0.001) |
| Obesity | 3.853 (<0.001) | 12.391 (<0.001) |

Odds ratio of increasing COVID-19 disease severity when comparing multiple factors. P-values are included in parentheses. For variants, all odds ratios are compared to Ancestral and for Race all odds ratios are in comparison to White.
