# Supplementary material for: Impact of SARS-CoV-2 variants and demographic factors on COVID-19 severity in pediatric patients
Source: BMC Infect Dis. 2026 Jul 17;26:1804. doi: 10.1186/s12879-026-13987-z (PMC13660046; doi:10.1186/s12879-026-13987-z)
Supplement: Supplementary file 10 [file 12879_2026_13987_MOESM9_ESM.docx]

**Estimated COVID-19 variant for each MIS-C patient.** Two months prior to admission date was used as an estimator for COVID-19 variant for each MIS-C patient. Vaccination availability is also plotted for each age group. Each line is drawn to intersect all possible variants and does not convey any quantitative information.
